# Supplementary material for: A study on a vehicle semi-active suspension control system based on road elevation identification
Source: PLoS One. 2022 Jun 24;17(6):e0269406. doi: 10.1371/journal.pone.0269406 (PMC9232168; doi:10.1371/journal.pone.0269406)
Supplement: Attachment — Submitted filename: responds to the reviewer#2s comments.docx [file pone.0269406.s002.docx]

Response to Reviewer 2 Comments

Dear Reviewers:

Thank you for your letter and for the reviewers' comments concerning our manuscript entitled "A Study on a Vehicle Semi-Active Suspension Control System Based on Road Elevation Identification" （ID:PONE-D-22-05626）.Those comments are all valuable and very helpful for revising and improving our paper, as well as the important guiding significance to our researches. We have studied comments carefully and made correction which we hope meet with approval. Revised portion are marked in yellow in the paper. The main corrections in the paper and the responds to the reviewer's comments are as flowing:

**Point 1:** Figure 2 does not clearly describe the physical meaning of nL, i.e., ‘relative pitch angle between the vehicle body and wheel’.

**Response 1:** We have improved Figure 2, aligned it with the vehicle coordinate system in Figure 4, and showed the pitching action of the vehicle body relative to the wheel. In the figure, the pitching angle nL is marked.

**Point 2:** In Figure 2, X0-Y0-Z0 is the vehicle body fixed coordinate system?

**Response 2:** Since we have modified Figure 2 and aligned it with the vehicle coordinate system in Figure 4, X0-Y0-Z0 in Figure 2 becomes the vehicle coordinate system with the vehicle centroid as the origin.

**Point 3:** LIDAR is a keyword of the paper. It is better to define this term at the beginning of the paper. Thus, the term of ‘laser LIDAR’ appeared in the context may be simplified as ‘LIDAR’.

**Response 3:** We have defined lidar at the beginning. Please check 'line 128 to line 130' in the revised document, and replace all the term of‘laser LIDAR’appeared in the context involved in the text with "LIDAR".

**Point 4:** In Equations (3) and (4), ‘z’ is used, but in the sentence below Equation (3), ‘Z’ is utilized. The principle of consistency needs to be followed.

**Response 4:** We have replaced 'Z' in the sentence below Equation (3) with 'z', please see 'Line 163' in the revised document.

**Point 5:** Below Equation (1), nL is defined as ‘Relative pitch angle between the vehicle body and wheel’, while below Equation (5), nL is redefined as ‘Disturbances caused by vertical bumps and other movements of the vehicle’. For a single symbol, double definitions need to be avoided.

**Response 5:** We have replaced the symbol "disturbance caused by vehicle vertical turbulence and other motion" under equation (5) with "pL" instead of "nL". Please see "line 168" in the revised document.

**Point 6:** It is not clear if vector components are introduced in Equations (6) and (7). Based on the second equation of equation set (7), it seems that all components on the equation should be scalars instead of vectors. If this is the case, the arrow signs need to be removed in order to avoid confusion.

**Response 6:** All components in equations (6) and (7) are changed from vector to scalar, and remove all potentially confusing the arrow signs in the paper.

**Point 7:** On page 8, it is stated: “… and the probability density of the measurement point can be approximated by a continuous Gaussian normal distribution function…”. It is not clear what is the base for this assumption.

**Response 7:**It is really true as Reviewer suggested that the paper is not clear what is the base for this assumption,and we have re-written this part according to the Reviewer's suggestion. Based on the uneven distribution of pulse signals emitted by lidar, we have supplemented the probability density assumption basis of measurement points in this paper. Please see 'lines 192 to 196' in the revised document.

**Point 8:** The vehicle body fixed coordinate system shown in Figure 4 should be consistent with that illustrated in Figure 2.

**Response 8:** We have adjusted the vehicle coordinate system in Figure 2 to make it consistent with the vehicle coordinate system in Figure 4, so the body coordinate system in Figure 4 has not been modified.

**Point 9:** As shown in Figure 4, a seven DOF vehicle model is generated in the study. It is not clear if the model is validated using experimental data or other methods.

**Response 9:** The seven DOF vehicle model established in this study can reflect the actual motion state of the vehicle. It is a classic model in the industry. The geometric parameters, mechanical parameters (stiffness and damping) and mass parameters (mass and moment of inertia) of the model are derived from the real vehicle data.

**Point 10:** It seems that only shock absorber damping is controlled during the operation of the suspension. If this is the case, the term of semi-active suspension should be used instead of active suspension.

**Response 10:** We have replaced all the term of active suspension in this paper with semi-active suspension.

**We appreciate for Reviewers warm work earnestly, and hope that the correction will meet with approval.**

**Once again, thank you very much for your comments and suggestions.**
